# Effectiveness of a school-based intervention using alternative sports to improve physical and motor skills in elementary school students (RENUÉVATE Project): study protocol for a randomized controlled trial

**DOI:** 10.3389/fpubh.2026.1817081

**Published:** 2026-05-21

**Authors:** Paz Pezoa-Fuentes, Mairena Sánchez-López, Eugenio Merellano-Navarro, Claudia Morales-Vejar, Sebastián Valenzuela-Díaz, Mirko Aguilar-Valdés, Exal Garcia-Carrillo

**Affiliations:** 1 Department of Physical Activity Sciences, Faculty of Education Sciences, Universidad Católica del Maule, Talca, Chile; 2 Center for Social and Health Research, Universidad de Castilla-La Mancha, Cuenca, Spain; 3 Faculty of Education, Universidad de Castilla-La Mancha, Ciudad Real, Spain; 4 Faculty of Education Sciences, Universidad Católica del Maule, Talca, Chile; 5 Education Consortium Program, Faculty of Education Sciences, Universidad Católica del Maule, Talca, Chile; 6 Department of Physical Activity Sciences, Universidad de Los Lagos, Osorno, Chile

**Keywords:** physical education and training, schools, motor skills, exercise, teaching, child

## Abstract

**Background:**

Physical inactivity and low enjoyment of physical education during childhood represent a significant public health problem. Although schools are a key setting for promoting active lifestyles, physical education instruction continues to focus largely on traditional sports, which may limit student participation and engagement. In this context, alternative sports emerge as an innovative and potentially more inclusive pedagogical strategy. Therefore, the objective of this study is to evaluate the effectiveness of an alternative sports intervention in the school setting, compared to standard physical education, on physical fitness, body composition, motor competence, and enjoyment of physical activity among elementary school students.

**Methods:**

A cluster-randomized controlled trial will be conducted among 11- to 12-year-old schoolchildren. Schools will be selected at random, and within each school, two classes from the same grade will be assigned to the intervention or control groups. The intervention will consist of an eight-week instructional unit (16 sessions) based on four alternative sports (flag football, ultimate frisbee, korfball, and kin-ball) taught using the Teaching Games for Understanding approach. The control group will continue with the regular physical education curriculum. Physical fitness, motor competence, and enjoyment of physical activity will be assessed as primary outcomes, while physical activity levels and body composition will be considered secondary outcomes. All variables will be assessed before and after the intervention. Primary analyses will follow the intention-to-treat principle using mixed-effects models to account for clustering (students within schools). The effects of the intervention will be estimated using the group × time interaction. Analyses will be performed using SPSS v28 and R v4.3.

**Discussion:**

This protocol addresses significant gaps in the literature on school-based interventions using alternative sports. It is expected that the experimental design, together with a structured intervention that can be replicated by teachers, will generate robust evidence on the physical, motor, and affective-motivational mechanisms associated with this pedagogical approach. The anticipated findings may serve as a basis for future curricular decisions and educational policies aimed at promoting physical activity and health during school-age years.

**Clinical trial registration:**

ClinicalTrials.gov, identifier NCT07313267.

## 1 Introduction

Along with body composition, physical fitness and motor competence in childhood are increasingly recognized as key indicators of health and important determinants of current and future physical activity (1, 2). Higher levels of physical fitness and better motor skills during childhood have been associated with better functional performance and greater participation in sports (3), as well as with more favorable health trajectories in later life (4). Late childhood (approximately ages 8–13) represents a particularly important period for consolidating fundamental motor skills and sport-related skills (5, 6), developing positive attitudes toward movement, and shaping sustained physical activity behaviors (7).

In the field of physical education, sport-based approaches constitute one of the most widely implemented pedagogical strategies for promoting physical activity, motor development, and social interaction in childhood (8). Sports offer structured and regulated environments that promote systematic motor practice, decision-making, and active participation, fundamental pillars for motor learning and the improvement of physical fitness (9). However, both the nature of the sport and the pedagogical model adopted significantly influence students’ levels of adherence and the effectiveness of learning outcomes (10).

In Chile, the school environment offers a strategic opportunity to promote physical activity and motor development on a large scale. Physical education, as part of the national educational framework, plays a fundamental role in students’ holistic development and in fostering active lifestyles, leadership, and self-care (11). However, participation in organized sports and extracurricular physical activities is not distributed equitably (12). Economic, cultural, and logistical barriers can limit access and sustained participation, particularly among children who do not identify with traditional sports models or who perceive themselves as less competent. Therefore, school-based approaches that prioritize participation, enjoyment, and inclusion can help reduce inequalities in physical activity opportunities.

Traditional sports in physical education typically include widely institutionalized activities, such as soccer, basketball, and handball, which are often characterized by a strong emphasis on technical performance, competition, and early specialization (13).

While these sports can be effective, they may also inadvertently limit the participation of students with perceived lower competence or little prior experience (13). In contrast, alternative sports are defined as non-traditional, adaptable, and often less institutionalized physical activities that prioritize inclusion, cooperation, and diverse motor experiences (14). These sports typically involve modified rules, mixed-gender participation, and a reduced emphasis on competition, which can foster a more inclusive and motivating learning environment (15). These practices promote students’ holistic development, increase motivation and participation in physical education classes (16), and foster equity, cooperation, and decision-making (16, 17).

It is important to note that alternative sports can also reduce structural and economic barriers, as they often require minimal specialized infrastructure and can be adapted to available school resources. This flexibility can facilitate broader implementation across diverse educational contexts, particularly in resource-limited settings, promoting students’ holistic development and fostering equity and cooperation among students (16, 17).

From a physiological perspective, participation in alternative sports can contribute to improvements in body composition through increased engagement in moderate-to-vigorous physical activity (18), higher participation rates, and a reduction in sedentary behavior during school hours (19). These factors are known to influence energy expenditure and can lead to favorable changes in fat mass and lean mass over time (20).

As for evidence regarding physical fitness and motor skills, this is much more limited and comes primarily from a few interventions focused on a single sport. Improvements have been reported in strength and power in disciplines such as padel (21) and street workout (18), in broader components of physical fitness such as endurance, strength, and speed through kin-ball (22); and, in some cases, improvements in motor skills compared to traditional physical education programs (23). However, there remains a lack of high-quality studies to determine the effects of systematic alternative sports programs implemented in schools on physical fitness, motor competence, and enjoyment among elementary school children. The RENUÉVATE project was designed to address this gap in the evidence.

Our hypothesis is that participants assigned to the intervention will show greater improvements than controls in key components of physical fitness (e.g., muscle strength, speed, and cardiorespiratory endurance), body composition (fat mass and fat-free mass), motor competence (including body and object control), and enjoyment of physical activity. Using a randomized controlled trial design and a comprehensive set of outcomes, RENUÉVATE will provide robust evidence on whether alternative sports can serve as an inclusive and engaging strategy within the school setting to promote physical and motor development, with potential implications for educational practice and early health promotion.

## 2 Objective

The aim of this study is to evaluate the effectiveness of an alternative sports program in a school setting, compared to standard physical education, on physical fitness, body composition, motor skills, and enjoyment of physical activity among elementary school students aged 11–12.

## 3 Materials and methods

### 3.1 Study design

**Figure 1:**
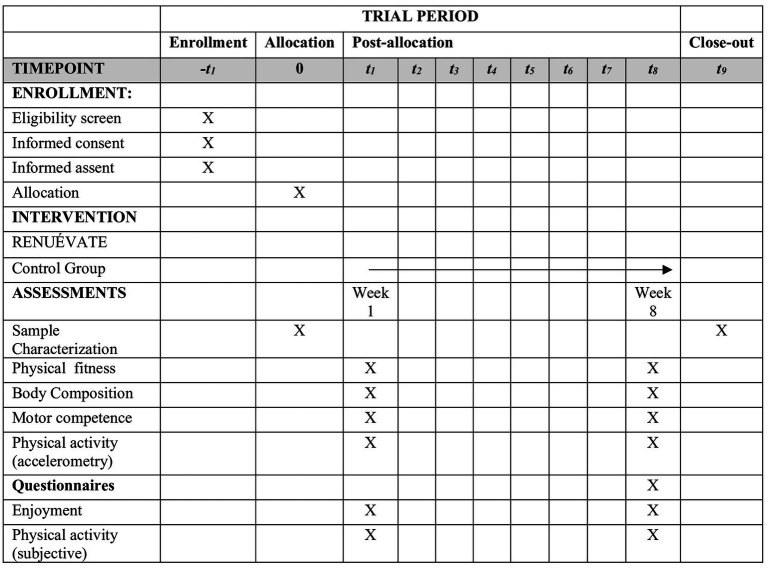
Standard protocol items: recommendations for intervention trials (SPIRIT) (24). −t_1_, school and participant enrollment; 0, group assignment and baseline assessments (weight, height); t_1_–t_8_, active intervention periods; assessments at t_1_ and t_8_; t_9_, follow-up assessments (weight, height).

The study adopts a cluster-randomized controlled trial (cRCT) design with repeated measures and parallel groups (three intervention groups and three control groups), following a quantitative approach. This protocol was developed in accordance with the SPIRIT guidelines for clinical trial protocols (24). The trial will be reported following the CONSORT statement and its extension for cRCTs (25). The overall study design and allocation process are illustrated in Figure 1.

Authorization to conduct the intervention will be obtained from the participating schools, and parents or legal guardians will provide written informed consent prior to student participation. Upon completion of the study, families will receive a summary report of their children’s assessment results.

### 3.2 Sample size

The trial is designed as a cRCT with the class group as the unit of randomization. The minimum clinically relevant difference in body fat percentage has been defined as 3 percentage points (*Δ* = 3), with an estimated standard deviation of 5. A significance level of *α* = 0.05 (two-sided) and a statistical power of 80% were assumed. An average group size of *n* = 35 students and a loss-to-follow-up/dropout rate of 15% were considered. Based on recent literature on randomized controlled trials (RCTs) in school-aged children, an intracluster correlation coefficient (ICC) of 0.03 was assumed (18).

**Figure 2:**
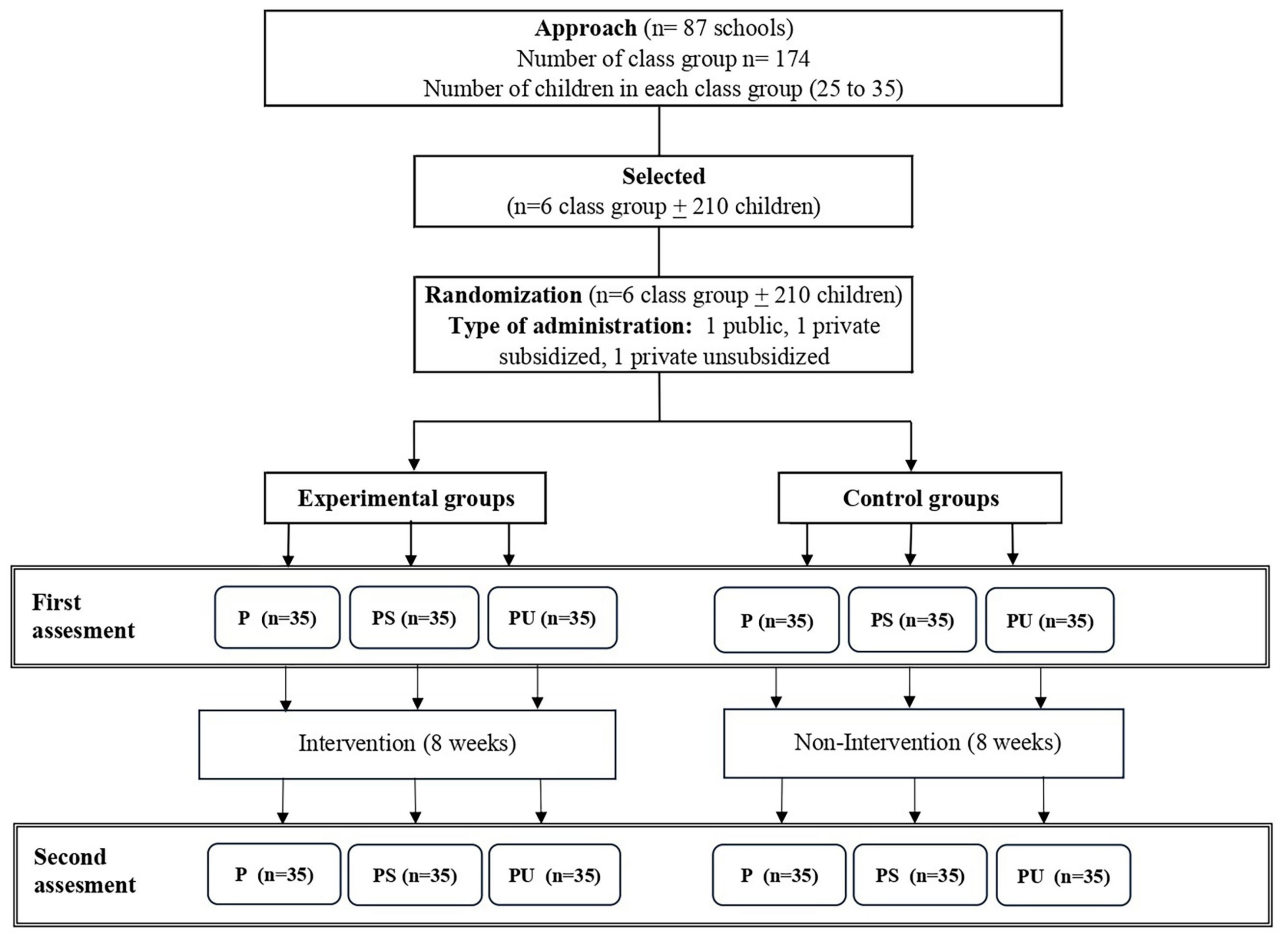
Study flowchart. P: Public administration; PS: Subsidized private administration; PU: Unsubsidized private administration.

With these assumptions, approximately 44 students per group are needed in a single trial. Adjusting for the cluster effect with an ICC = 0.03, the group size increases to approximately 88 students. Therefore, considering a 15% attrition rate, the final group size is approximately 104 students. To achieve this with groups of an average size of 35, 3 courses/groups per cohort (6 groups in total) will be required, implying 210 enrolled students (105 in the intervention group/105 in the control group). The flow of participants throughout the study is summarized in Figure 2.

Although body composition is not the primary outcome, it was selected as the reference variable for sample size calculation due to its high sensitivity and the existence of well-established thresholds of clinical relevance in the academic literature (26, 27). As it is a variable with greater inter-subject variability in short- to medium-term interventions, the sample size derived from it acts as a conservative estimate. This approach ensures robust statistical power (>80%) to detect significant effects not only in body composition but also in secondary variables with lower sample size requirements, such as motor competence or enjoyment (28).

### 3.3 Randomization and blinding

Randomization will be conducted at the school and class levels. Urban schools in the city of Talca, Chile, will be randomly selected using a computer-generated sequence. Three schools will be included, representing different administrative types within the Chilean education system: a public school, a subsidized private school, and a private school.

Within each participating school, two intact classes will be included. One class will be randomly assigned to the intervention group (IG) and the other to the control group (CG), resulting in parallel groups within the same educational setting.

Due to the nature of the school-based intervention, it will not be possible to blind participants, teachers, and intervention staff. However, data management and statistical analyses will be conducted using anonymized datasets to minimize potential bias.

To ensure treatment fidelity and minimize experimenter bias, the intervention will be implemented by an external researcher who is specialized and specifically trained in the study protocol (29). In contrast, the control group will maintain its usual curriculum as led by the school’s regular teacher. This design not only allows for rigorous standardization of the independent variable but also ensures that the control group reflects real-world conditions, thereby preserving the ecological validity of the study in naturalistic school settings (30, 31).

### 3.4 Inclusion criteria

Students aged 11–12 who are in the sixth grade of elementary school and whose parents provide written informed consent for their participation.

### 3.5 Exclusion criteria

Students with a medical condition that prevents them from participating in alternative sports or those with a cognitive disability will be excluded. These criteria are used because the effect of the intervention on the study variables (physical fitness, motor skills, physical activity level, and enjoyment) depends on the students’ physical and psychological readiness to perform the various motor tasks.

### 3.6 Recruitment, participants, and assignment

Educational institutions will be randomly selected and invited to participate through an initial email contact with school principals, followed by a telephone follow-up 1 week later. Subsequently, a member of the research team will meet with interested principals to explain the study’s objectives, procedures, and participation requirements. Once institutional approval is obtained, informational meetings will be scheduled with teachers and students at the selected schools.

Each participating school will provide two class groups, with one group assigned to the IG and the other to the CG. Schools that agree to participate will receive written information in plain language, along with informed consent forms for parents or legal guardians. Recruitment will take place over a two-month period in 2026 at three elementary schools in the city of Talca, Chile.

Following institutional approval, detailed information about the study will be provided to students in the selected classes. Only students whose parents or legal guardians provide written informed consent and who give their assent will be included in the data collection. Participation will be voluntary, and students may withdraw from the study at any time without academic consequences.

### 3.7 Intervention

#### 3.7.1 Intervention group (IG)

**Figure 3:**
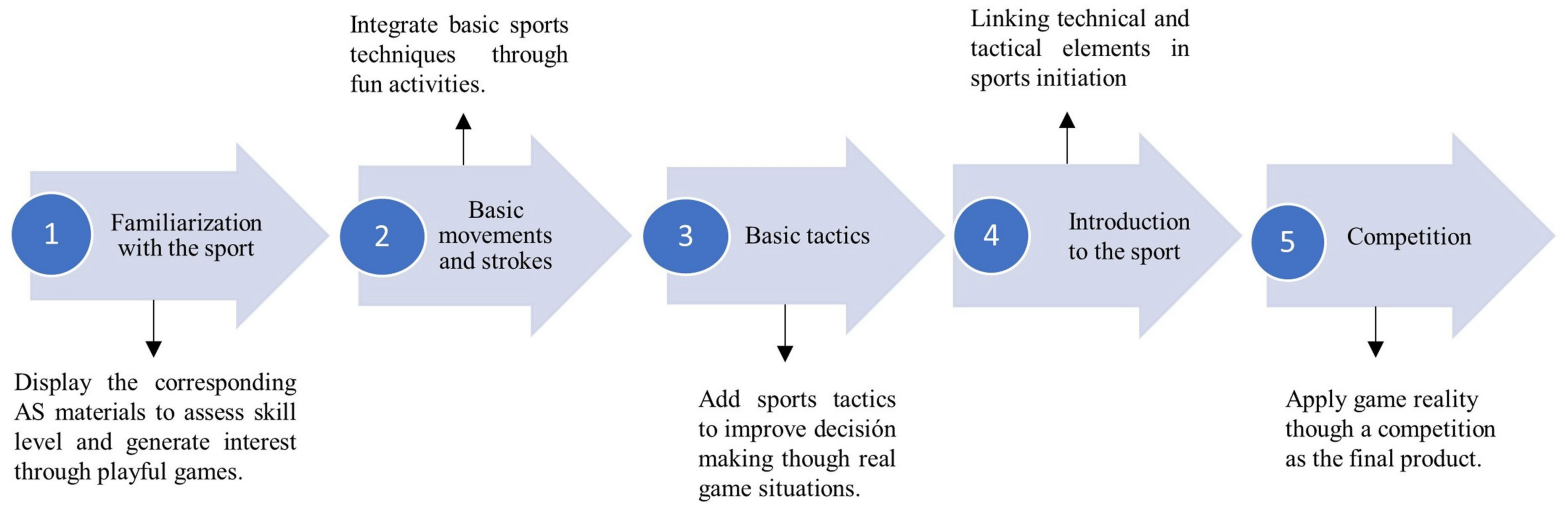
Diagram of the methodological sequence for teaching alternative sports, adapted from Morales-Campo et al. (15), and modified by the authors.

The IG will receive a pedagogical intervention based on the Teaching Games for Understanding (TGfU) model (14), specifically adapted for teaching alternative sports (32) (Figure 3).

To maximize treatment fidelity and minimize inter-instructor variability, all sessions will be conducted by an external physical education instructor who has been thoroughly trained in the research protocol. This methodological strategy ensures standardized application of the pedagogical model, regardless of the usual teaching practices at each participating school.

The intervention consists of four teaching units, each lasting 2 weeks, focused on: flag football, ultimate frisbee, korfball, and kin-ball. These disciplines were selected for their low requirement for prior specialization, their potential for developing motor skills, and their ability to promote gender equity and social inclusion (33–35).

The pedagogical progression will follow a structure of increasing complexity in three phases: (1) initiation through simplified rules and general game situations; (2) development of tactical awareness and motor execution through modified games; and (3) integration into real game scenarios with an emphasis on decision-making. For example, in ultimate frisbee, the progression moves from basic throwing and catching skills in dynamic contexts to situations of numerical superiority in confined spaces, culminating in full-format games where intelligent use of space and offensive-defensive transitions are prioritized.

#### 3.7.2 Control group (CG)

The CG will participate only in the baseline and post-intervention assessments, maintaining its regular physical education curriculum led by its regular teacher, with no changes to the existing lesson plans or teaching methodology. The sessions will be governed by the National Curriculum Standards currently in effect in Chile, specifically in the development of Unit 2: Individual and Team Games and Sports (11).

The curriculum offered in the CG will include traditional sports (i.e., basketball, soccer, and handball) using a traditional technical-pedagogical approach, characterized by an emphasis on the acquisition of fine motor skills and basic tactical patterns. Lessons will predominantly employ direct instruction strategies (command style), based on teacher demonstration, repetitive practice of tasks (drills), and prescriptive corrective feedback. Equivalence in activity volume (frequency and duration of sessions) between the IG and the CG will be ensured to guarantee group comparability.

It should be noted that the design of this study does not aim to contrast ‘effective’ teaching with ‘ineffective’ teaching. On the contrary, it is recognized that well-structured traditional Physical Education can promote motor learning through skill progression (36). Therefore, the objective of this comparison is to determine whether a comprehensive teaching model (TGfU) integrated with alternative sports provides incremental or superior benefits in motor competence and health compared to the standard pedagogical practice (usual care) of the educational system (37).

#### 3.7.3 RENUÉVATE program

Each session will last 90 min and will follow a standardized structure consisting of three clearly differentiated phases:

##### 3.7.3.1 Warm-up (15 min)

The initial phase will include general joint mobility exercises and sport-specific activities designed to prepare students for the corresponding alternative sport. Activities will be performed at a low to moderate intensity, equivalent to 50–60% of maximum heart rate (HRmax) and a rate of perceived exertion (RPE) between 9 and 11. The objective of this phase is to progressively activate the musculoskeletal and cardiovascular systems, reduce the risk of injury, and facilitate preparation for learning.

##### 3.7.3.2 Main part (65 min)

This phase will focus on practicing the alternative sport through modified game situations and technical-tactical progressions, in line with the TGfU approach. Tasks will be designed to promote decision-making, game understanding, and motor execution, maintaining a moderate to vigorous intensity (65–80% of HRmax; RPE 12–15) (4).

Active recovery periods will be incorporated between activities to promote recovery and continuity of practice. To validate the intensity achieved, wrist-based heart rate monitors will be used on a subsample comprising 25% of the participants.

##### 3.7.3.3 Cool-down (10 min)

The session will conclude with low-intensity recovery activities, including relaxation exercises, stretching, and a brief group reflection on motor learning and the values addressed during the session. This phase will be conducted at a light intensity, below 50% of HRmax, with a RPE ≤ 9.

### 3.8 Implementation, intervention fidelity, and teacher training session

Before the start of the intervention, the physical education teacher assigned to the IG will receive a specific training session delivered by the research team. This training will last approximately 45–60 min and will cover the theoretical foundations of the RENUÉVATE program, the TGfU methodology applied to alternative sports, the intervention’s objectives, the organization of the sessions, and instructions for the proper implementation and evaluation of the program. Once the training is complete, the necessary instructional and support materials for carrying out the intervention will be provided. To ensure proper implementation and adherence to the RENUÉVATE intervention protocol, a member of the research team will supervise the initial phases of the process. During the first weeks of the intervention, the researcher will provide technical and methodological support to the physical education teacher responsible for the intervention group, answering their questions, adjusting activities as necessary, and providing ongoing feedback.

In the initial phase, the teacher will conduct the sessions according to the established plan for each alternative sport, while the researcher will observe the classes and offer suggestions aimed at optimizing the application of the TGfU methodological approach adapted to alternative sports. Subsequently, the teacher will conduct the sessions independently, maintaining ongoing communication with the research team to address any issues that arise during the program’s implementation.

The fidelity of the intervention will be assessed through direct observation using a standardized checklist based on the key components of the TGfU model (e.g., emphasis on modified play, tactical challenge questions, and transitions). An external observer will evaluate 20% of the sessions at random. In addition, a session log self-reported by the instructor will be used.

Decision-making based on fidelity will be structured as follows: if the fidelity score falls below 80% in a session, an immediate retraining session for the instructor will be initiated prior to the next teaching unit. If deviations persist, data from that specific group will be analyzed using “intention-to-treat” and “per-protocol” analyses to assess the impact of the instructional dose on final outcomes.

### 3.9 Procedures, outcome measures, and data collection

Assessments will be conducted at two time points: at baseline, prior to the start of the intervention (Session 1), and at the end of the program (Session 16). At both time points, anthropometric measurements, physical fitness, motor competence, objectively and subjectively assessed physical activity levels, and enjoyment of physical activity will be collected.

Data collection will be carried out by a team of trained research assistants, who will receive prior instruction from the research team through specific training sessions designed to ensure the correct application of the assessment instruments and the standardization of procedures. The evaluators will have no prior relationship with the students, and their role in the study will be limited exclusively to data collection, in order to minimize any potential bias in the evaluation.

#### 3.9.1 Primary outcome

##### 3.9.1.1 Motor competence

Motor competence will be the primary outcome of the study and will be assessed using the MOBAK 5–6 (Motorische Basiskompetenzen) battery, validated for fifth- and sixth-grade elementary school children (38). This instrument assesses basic motor competence across two dimensions: self-movement (balance, rolling, jumping, and moving) and object movement (throwing, catching, bouncing, and dribbling).

Each dimension includes four motor tasks, which are scored dichotomously (0 = task not achieved; 1 = task achieved according to established criteria). Scores for each dimension range from 0 to 4 points, and the total score ranges from 0 to 8 points, with higher values indicating a higher level of motor competence.

#### 3.9.2 Secondary outcomes

##### 3.9.2.1 Physical fitness

Physical fitness will be assessed using standardized field tests. Cardiorespiratory fitness will be measured using the 6-min walk test, which is used to estimate maximum oxygen consumption (VO₂max) (39). Lower limb muscle strength will be assessed using the standing long jump test, while upper limb muscle strength will be assessed using a handgrip strength test with a dynamometer (Camry EH101), following validated protocols (40).

##### 3.9.2.2 Body composition and morphological variables

Body composition will be estimated using an eight-electrode tetrapolar bioelectrical impedance analysis (InBody 570®) (41) to obtain indicators of fat mass and muscle mass.

Morphological variables will include body weight, height, body mass index (BMI), and waist circumference; measurements will be taken by an anthropometrist accredited by the International Society for the Advancement of Kinanthropometry (ISAK) Level II (42). The ISAK protocol consists of a standardized measurement technique that ensures reproducibility by first marking specific anatomical landmarks (e.g., acromial, radial, iliac) and taking duplicate measurements (or triplicate if the difference exceeds 5%). A calibrated anthropometric kit (skinfold caliper, metal tape measure, and anthropometer) will be used to ensure that the technical error of measurement (TEM) is less than 1% for waist circumference.

##### 3.9.2.3 Physical activity level (target)

Physical activity levels will be objectively assessed using triaxial accelerometry (ActiGraph wGT3X-BT, Pensacola, FL, USA) (43). Following the RENUÉVATE Specific Monitoring Protocol, the devices will be placed on the right hip using an adjustable elastic belt, ensuring their stability during multidirectional movements.

In accordance with the study’s central objective, to evaluate the pedagogical efficacy of alternative sports, the accelerometers will be used exclusively during Physical Education sessions (90 min). The research team will personally supervise the placement at the start and removal at the end of each class during the 8-week intervention. This methodological decision allows for rigorous control of the physical activity dose strictly linked to the pedagogical model (TGfU vs. Traditional), minimizing statistical noise from extracurricular activities.

The devices will be configured with a sampling rate of 30 Hz, and data will be processed in 15-s epochs, optimizing the capture of the intermittent nature of children’s movement (44). Non-use time will be defined as ≥20 consecutive minutes of zero counts. To ensure data validity, a session will be considered valid if it records at least 80% of the total class time, thereby ensuring an accurate representation of motor behavior during the intervention.

The intensity of physical activity will be categorized using the intensity thresholds (cut-off points) validated by Evenson et al. (45) for the pediatric population, due to their high accuracy in classifying activity levels in school settings. Data will be classified according to the following counts per minute (cpm) ranges: Sedentary: 0–100 cpm; Light physical activity (LPA): 101–2,295 cpm; Moderate physical activity (MPA): 2296–4,011 cpm; Vigorous physical activity (VPA): ≥4,012 cpm.

The primary movement variable, moderate-to-vigorous physical activity (MVPA), will be obtained by summing the time recorded in the moderate and vigorous categories. This indicator will allow us to quantify compliance with international health recommendations and determine the actual impact of the pedagogical model on students’ energy expenditure during class.

##### 3.9.2.4 Physical activity level (self-reported)

The Youth Activity Profile (YAP-LA) (46) is a self-report questionnaire consisting of multiple items with responses on a five-point Likert scale that assesses physical activity and sedentary behavior during school and after-school hours over the previous 7 days. Composite scores are calculated according to the standardized scoring protocol, where higher scores indicate higher levels of physical activity.

##### 3.9.2.5 Enjoyment of physical activity

Enjoyment of physical activity will be assessed using the Physical Activity Enjoyment Scale (PACES), validated in school-aged populations (47). The scale consists of 16 items rated on a five-point Likert scale that assess positive and negative affective responses associated with participation in physical activity. Negatively worded items will be reverse-coded, and a total score will be calculated, where higher values indicate greater enjoyment.

##### 3.9.2.6 Sociodemographic variables and covariates

Sociodemographic variables will be collected via a questionnaire completed by parents or legal guardians, including educational level, household income, and family structure. These variables will be used to characterize the sample and will be considered covariates in the statistical analyses.

### 3.10 Reliability thresholds and control of technical error of measurement

Prior to the start of fieldwork, a pilot study will be conducted with approximately 10% of the total sample to assess the reliability of all anthropometric, physical, and questionnaire-based measurements included in the study. This pilot phase will allow for the verification of measurement accuracy and the standardization of assessment procedures prior to data collection.

For continuous variables, the following reliability indicators will be calculated: absolute TEM, relative TEM (%TEM), coefficient of variation (CV%), and intraclass correlation coefficient (ICC) with 95% confidence intervals. Acceptable reliability thresholds will be defined as follows: %TEM ≤ 0.5% for height and body mass, and ≤1% for circumferences (intra-rater reliability). For performance-based physical fitness tests, a CV ≤ 3% will be required for speed and agility tests, and a CV ≤ 5% for jump-based tests.

For self-report instruments, test–retest reliability will be assessed using the ICC, calculated over a retest interval of 7 to 14 days, with ICC values ≥0.70 considered acceptable. Internal consistency will be assessed using Cronbach’s alpha, with values ≥0.70 indicating adequate reliability.

If any rater fails to meet the predefined reliability criteria, they will be provided with additional training, and the measurements will be repeated until acceptable thresholds are reached. All reliability indicators (TEM, %TEM, CV%, and ICC with 95% confidence intervals) will be included in the final publication, and sensitivity analyses will be performed when appropriate.

#### 3.10.1 Quality assurance of measurements

All anthropometric and physical fitness assessments will be performed by assessors who have been previously trained and standardized by the research team. To ensure measurement quality during the main study, inter-rater reliability will be assessed in a random subsample comprising approximately 10% of the participants.

Inter-rater reliability will be estimated using ICC and TME. Acceptable thresholds will be defined as a TME < 2% for anthropometric variables and <5% for physical fitness tests, in accordance with established methodological recommendations (48). These procedures will ensure consistency among assessors and maintain the quality and reproducibility of measurements throughout the data collection process.

### 3.11 Ethics and dissemination: research ethics approval, consent, and confidentiality

This study protocol was designed and will be conducted in accordance with the ethical principles established in the Declaration of Helsinki and with current ethical regulations for research in physical activity and health sciences involving school-aged populations. The study was reviewed and approved by the Scientific Ethics Committee of the Universidad Católica del Maule, Chile (No. 112/2025, approved on July 3, 2025).

Before the intervention begins, the parents or legal guardians of participating students will receive detailed and understandable information about the study’s objectives, the procedures involved, the potential benefits and risks, and the voluntary nature of participation. Next, written informed consent authorizing the students’ participation will be obtained. In addition, students will provide their informed consent, expressing their willingness to participate in the study, prior to the administration of any assessment or intervention.

Confidentiality and the protection of personal data will be ensured throughout all phases of the study. The collected data will be coded and stored securely, and access will be restricted exclusively to the research team. Under no circumstances will the data be used for purposes other than the scientific and academic objectives defined in the protocol. The study results will be analyzed and reported in aggregate form, ensuring the confidentiality and anonymity of all participants. The information obtained will be used solely for scientific, academic, and knowledge-advancement purposes in the field of physical education.

### 3.12 Data management, access, and dissemination policy

Data collection will be conducted at the participating educational institutions, following the standardized procedures defined in the study protocol. This process will be carried out by a team of trained research assistants who will be responsible for administering the assessment instruments and recording the initial data.

The collected data will be transferred to the principal investigator and the co-investigator, who will oversee the review, organization, and secure storage of the data. All information will be stored in password-protected digital databases, and a backup copy will be saved in a secure Google Drive folder, with access restricted exclusively to the research team. Subsequently, the cleaned datasets will be exported to statistical software for analysis, and access to these data will be limited to the researchers responsible for the study.

Once the anonymization procedures are complete, the de-identified datasets may be shared with collaborating researchers or peer reviewers upon reasonable request and solely for scientific purposes, through controlled access to a digital repository, always ensuring the confidentiality and privacy of the participants.

The study results will be disseminated both nationally and internationally. National dissemination will include academic and scientific outreach activities, such as seminars, webinars, and institutional educational platforms. Internationally, the results will be presented at scientific conferences and submitted for publication in indexed, peer-reviewed journals related to physical activity, health, and education.

### 3.13 Statistical analysis

#### 3.13.1 Descriptive analysis

The baseline characteristics of the participants will be described using descriptive statistics. Continuous variables will be summarized as means and standard deviations or medians and interquartile ranges, as appropriate, after assessing the data distribution using the Kolmogorov–Smirnov test and visual inspection. Categorical variables will be presented as frequencies and percentages.

#### 3.13.2 Primary analysis (intention-to-treat)

The primary analysis will be conducted according to the intention-to-treat (ITT) principle, including all participants as they were originally assigned, regardless of their adherence to the intervention. To estimate the effects of the RENUÉVATE intervention, mixed-effects regression models will be used to adequately account for the cRCT design (students nested within schools).

The effects of the intervention will be assessed through the group × time interaction, which represents the differences between groups in changes from baseline (Session 1) to post-intervention (Session 16). For continuous outcomes, including motor skills, physical fitness, enjoyment, physical activity levels, and body composition, linear mixed-effects models will be applied. For dichotomous outcomes, where applicable, generalized linear mixed models (GLMM) with a logit link function will be used.

All models will include group (intervention vs. control), time (baseline and post-intervention), and their interaction as fixed effects. Age and sex will be included as covariates. A random intercept will be specified for school to account for intraclass correlation within clusters.

#### 3.13.3 Sensitivity analysis (per protocol)

A per-protocol sensitivity analysis will be conducted, including only participants who attend at least 85% of the intervention sessions and complete both measurement points. The same mixed-effects modeling approach described for the ITT analysis will be applied to assess the robustness of the findings.

Patterns and proportions of missing data will be examined prior to analysis. When missing data exceed 5%, multiple imputations will be performed using chained equations under the assumption that data are missing at random. The imputation model will include all variables used in the primary analyses.

#### 3.13.4 Effect estimates and statistical inference

Results will be presented as adjusted between-group differences in change, with corresponding 95% confidence intervals (CI). Statistical significance will be set at *p* < 0.05 (two-sided). Standardized effect sizes will be calculated using Cohen’s d, based on adjusted estimates derived from mixed-effects models, to facilitate interpretation of the magnitude of the intervention effects.

All statistical analyses will be performed using SPSS version 28.0 (IBM Corp., Armonk, NY, USA) and R version 4.3, employing the lme4 package for mixed-effects modeling and the GGIR package for processing physical activity data derived from the accelerometer.

## 4 Discussion

The available evidence on physical education interventions in schools has largely focused on traditional sports and programs aimed at increasing physical activity levels, paying less attention to the integrated development of physical fitness, motor skills, and enjoyment during the early school years (49). Studies incorporating alternative sports as a central component of the intervention remain scarce, especially those designed with rigorous experimental methodologies and with the potential for replication by teachers in real school settings (50, 51).

In this context, the RENUÉVATE project aims to address this gap by designing and evaluating a structured school-based intervention based on a teaching unit that integrates four alternative sports, delivered by physical education teachers and evaluated via a cluster-randomized controlled trial (RCT). The protocol was developed in accordance with the SPIRIT recommendations for clinical trial protocols (21), and the study results will be reported following the CONSORT guidelines (25), including the extension for cluster trials. In this way, the study aims to generate methodologically sound evidence on the potential effects of alternative sports on physical, motor, and psychosocial outcomes in 11- to 12-year-old schoolchildren.

From a physiological perspective, interventions based on alternative sports are expected to lead to improvements in physical fitness through the alternation of moderate- to vigorous-intensity exertion, a characteristic of sports such as flag football and ultimate frisbee (32, 52). This pattern of intermittent exertion has been shown to be effective in stimulating adaptations in cardiorespiratory fitness, strength, and body composition in children, even in relatively short-term programs when the structure of the sessions is well-defined (53–55).

Similarly, the organization of sessions prioritizes a high volume of motor practice, which can translate into a greater effective physical activity load during class time. The inclusion of objective measures of physical activity using accelerometry will allow for the description and monitoring of the actual dose of the intervention, which will strengthen the interpretation of results in relation to the intensity achieved. From a motor development perspective, the selected alternative sports are characterized by requiring a wide range of motor skills, such as throwing, catching, multidirectional locomotion, jumping, and hand-eye coordination, within dynamic and constantly changing game contexts. These demands may contribute to improvements in general motor competence, a key aspect during the final stage of elementary school that is closely associated with future adherence to physical activity (56, 57). Systematic exposure to diverse, non-stereotypical motor tasks could particularly benefit students with limited experience in traditional sports, reducing barriers to participation and fostering more inclusive learning trajectories.

On an affective and motivational level, the intervention is grounded in the novelty, cooperation, and inclusivity inherent in sports such as kin-ball and korfball. These sports promote adapted rules, equitable participation, and collective goals, which can foster a task-oriented motivational climate, increasing enjoyment and intrinsic motivation toward physical activity (56, 58). Enjoyment of physical activity is a key determinant of sustained participation over time, and its assessment as a primary outcome in this protocol reflects the need to consider not only how much students move, but also how they experience motor participation in physical education classes.

Regarding the design of RENUÉVATE, it was developed to facilitate curricular integration by aligning with the usual instructional time allocated to physical education and using accessible material resources typically available in schools, emphasizing that no special infrastructure is required for its implementation. Furthermore, the detailed structure of the teaching unit and the TGfU-based approach (provided by the teacher) aim to ensure fidelity of implementation and promote transferability to other educational settings, including those with limited infrastructure or financial resources. The project seeks to support teaching practice and align with the school curriculum, particularly in Latin American contexts, where evidence on alternative sports remains very limited.

Among the protocol’s main methodological strengths is the use of a cluster-randomized experimental design, suitable for the school setting, along with an ITT analysis approach that helps preserve the study’s internal validity. The inclusion of objective measures (accelerometry), the assessment of multiple domains (physical, motor, and psychosocial), and the standardization of sessions through a detailed manual enhance the quality and replicability of the project. Furthermore, quality control of measurements and adjustment for the hierarchical structure of the data using appropriate statistical models will contribute to obtaining more precise estimates of the intervention’s effects.

Among the limitations of this protocol, the duration of the intervention (8 weeks) may be insufficient to consolidate long-term changes, as suggested by longitudinal studies on the stability of physical activity habits from childhood (59, 60). Furthermore, variability in implementation by teachers and potential cross-group contamination represent risks inherent to school-based studies. However, these limitations will be addressed through the standardization of sessions, monitoring of attendance, ITT analyses, and sensitivity and analyses conducted according to the protocol. Loss to follow-up will be managed using appropriate statistical strategies for missing data.

It is important to note that, if the intervention proves effective, the RENUÉVATE project could contribute to decision-making in education and public health by supporting the incorporation of alternative sports as a valid strategy for improving physical fitness, motor skills, and enjoyment of physical activity during school-age years. Furthermore, the protocol can serve as a model for future school-based interventions aimed at promoting active lifestyles in an inclusive, sustainable, and pedagogically coherent manner.

From a public health perspective, this study is particularly relevant, as it evaluates a scalable, school-based intervention that can be integrated into existing educational systems without requiring substantial additional resources. By leveraging physical education as a universal platform, the RENUÉVATE program has the potential to reach large populations of children and contribute to early prevention strategies targeting physical inactivity, motor delays, and associated health risks. This aligns with global public health priorities focused on promoting active lifestyles from an early age.

## 5 Conclusion

This protocol describes a structured and organized school-based intervention using alternative sports, aimed at evaluating its effects on physical, motor, and affective-motivational outcomes in elementary school students. The use of a randomized experimental design addresses the need to strengthen methodological rigor in research on physical education and school health, thereby maximizing internal validity and improving the transferability of findings to real-world educational settings.

The relevance of this study lies in its potential contribution to a still underdeveloped field of research, characterized by a predominance of interventions focused on traditional sports. In this regard, the RENUÉVATE project seeks to generate evidence that improves understanding of the role of alternative sports as a pedagogical strategy within the physical education curriculum, while providing useful information for the design of future interventions, support for teacher training, and decision-making regarding policies for the promotion of educational and school health.
